# Prognostic Significance of Malnutrition Indices in ST-Elevation Myocardial Infarction: A Comparative Analysis of CONUT and PNI Scores After Primary PCI

**DOI:** 10.3390/diagnostics16040573

**Published:** 2026-02-14

**Authors:** Fatma Can, Gönül Zeren, Tülay Bayram Gürkan, Zeynep Ece Demirbaş

**Affiliations:** 1 Department of Cardiology, Dr. Siyami Ersek Thoracic and Cardiovascular Surgery Training and Research Hospital, Istanbul 34668, Turkey; drftmcan@yahoo.com.tr (F.C.); gonulzeren@hotmail.com (G.Z.); drtulaybayram@hotmail.com (T.B.G.); 2 Department of Internal Medicine, Dr. Siyami Ersek Thoracic and Cardiovascular Surgery Training and Research Hospital, Istanbul 34668, Turkey

**Keywords:** Controlling Nutritional Status Monitor (CONUT), Prognostic Nutritional Index (PNI), ST-elevation myocardial infarction, primary percutaneous coronary intervention, malnutrition

## Abstract

**Background:** The purpose of this study was to compare the prognostic value of Controlling Nutritional Status (CONUT) and Prognostic Nutritional Index (PNI) scores for predicting in-hospital mortality among patients who presented with ST-segment elevation myocardial infarction (STEMI) and received primary percutaneous coronary intervention (P-PCI). **Methods:** This retrospective cohort study comprised 4599 STEMI patients who received P-PCI. The primary outcome was described as in-hospital mortality. Multivariable logistic regression analysis was performed to determine the association between in-hospital mortality and CONUT and PNI scores. Model performance and goodness-of-fit measures were used for comparison. **Results**: In-hospital mortality rate was 5.7% (*n* = 261). Patients who died during the index hospitalization were older and more likely to have diabetes, prior myocardial infarction (MI), revascularization history, longer total ischemic time, no-reflow and reduced left ventricular ejection fraction (LVEF). According to body mass index (BMI) categories, moderate-to-severe malnutrition was observed in approximately 17–21% and 8–10% of patients according to the CONUT and PNI scores, respectively. Both CONUT and PNI scores were significantly associated with in-hospital mortality. However, the model incorporating CONUT demonstrated superior goodness of fit and higher discriminative performance compared with the model incorporating PNI. **Conclusions:** Among patients with STEMI, moderate-severe malnutrition was present in nearly 10% and 20% when evaluated using PNI and CONUT, respectively. The CONUT score demonstrated superior predictive performance for in-hospital mortality compared with PNI.

## 1. Introduction

Coronary artery disease (CAD) has become a major global health concern due to its rapidly increasing prevalence [1,2]. In-hospital mortality in acute coronary syndromes (ACS) has declined from 30% to 3–8% over the past two decades, primarily due to advances in medical and pharmaco-invasive therapies [1,3]. Despite these advances in percutaneous intervention and new antiplatelet therapy choices, patients with ST-segment elevation myocardial infarction (STEMI) continue to be at high risk.

Malnutrition, irrespective of body mass index (BMI), is common in ACS patients and is associated with a poor outcome, regardless of the malnutrition assessment index used [4,5,6]. Both obesity and malnutrition increase the risk of cardiovascular disease and related mortality in the general population [7,8,9,10,11].

Many clinical, demographic, and laboratory factors, as well as risk scores, have been associated with hospitalization, adverse effects, and mortality in patients with ACS. Several studies have evaluated the prognostic value of either the Prognostic Nutritional Index (PNI) or the Controlling Nutritional Status (CONUT) score in patients with ACS, demonstrating their association with adverse outcomes and mortality [12,13,14]. However, few studies have directly compared these two nutritional indices within the same STEMI population [4,15,16]. These scores are objective markers of both malnutrition and immunological status [17]. For patients with ACS, there is an unmet need to investigate the prognostic influence of malnutrition on short-term mortality.

As a result, we aimed to assess and compare the prognostic significance of CONUT and PNI scores for predicting in-hospital mortality among STEMI patients who underwent primary percutaneous coronary intervention (P-PCI).

## 2. Methods

Patients who presented to our institution with STEMI and underwent P-PCI between January 2013 and January 2018 were retrospectively screened. Demographic, clinical, and laboratory data were retrieved from the hospital’s electronic medical records. ST-segment elevation myocardial infarction and the indication for P-PCI were defined according to current European Society of Cardiology guidelines [18]. Inclusion criteria were as follows:

1. ST-segment elevation of ≥1 mm in at least two contiguous leads (≥2 mm in leads V1–V3) or new-onset left bundle branch block on surface electrocardiography, accompanied by chest pain.
2. Treatment with P-PCI. (P-PCI was performed in patients presenting within 12 h of symptom onset, or up to 24 h in the presence of ongoing ischemia or hemodynamic instability. Symptom onset time was defined as the time of first chest pain suggestive of myocardial ischemia.)

Exclusion criteria were as follows:

1. Missing key clinical or laboratory parameters required for analysis.
2. Active malignancy.
3. End-stage renal disease or ongoing hemodialysis treatment.
4. Evidence of active infection at presentation.
5. Conditions not consistent with type 1 ST-segment elevation myocardial infarction, including Takotsubo syndrome and other STEMI-mimicking conditions.

This study was approved by the Hamidiye Scientific Research Ethics Committee of the University of Health Sciences (Türkiye) (Approval No: 23/523; Meeting No: 2023/16; Date: 1 September 2023). The study was conducted in accordance with the ethical principles of the Declaration of Helsinki. Since this was a retrospective study, the requirement for informed consent was waived.

**Figure 1:**
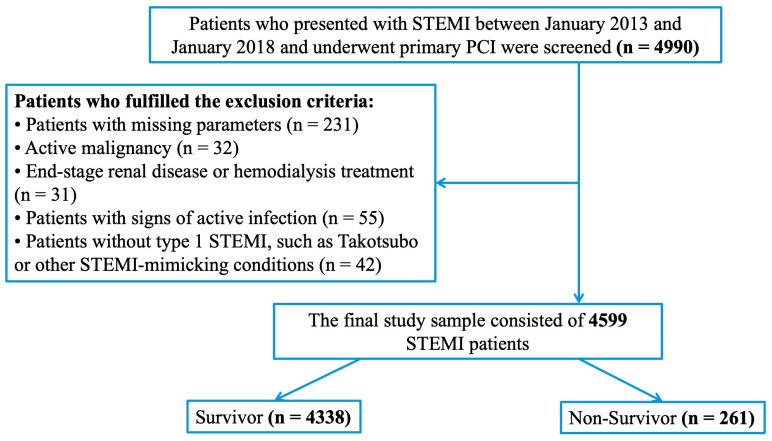
Flowchart of patient inclusion and exclusion criteria. PCI, percutaneous coronary intervention; STEMI, ST-segment elevation myocardial infarction.

The flow diagram of patient selection and inclusion criteria is presented in Figure 1. After applying the exclusion criteria, a total of 4599 patients with STEMI who underwent P-PCI were included in the final analysis.

### 2.1. Malnutrition Screening Tools

BMI was calculated as weight (kg) divided by height squared (m^2^). Patients were categorized as underweight (<18.5 kg/m^2^), normal weight (18.5–24.9 kg/m^2^), overweight (25.0–29.9 kg/m^2^), or obese (≥30 kg/m^2^).

CONUT score, developed by Ulibarri et al. in 2005, is derived from three routinely measured laboratory parameters: serum albumin concentration, total lymphocyte count, and total cholesterol level [19]. Each variable was assigned a specific score according to previously established cut-off ranges, and the sum of these points yielded a total score from 0 to 12. Patients were categorized as having normal nutritional status (0–1), mild (2–4), moderate (5–8), or severe (9–12) malnutrition.

The PNI was determined using the formula: (10 × serum albumin [g/dL]) + (0.005 × total lymphocyte count [mm^3^]) [10]. Lower PNI values indicate poorer nutritional and immune status. For descriptive purposes, patients were classified as having normal nutritional status (PNI > 45), mild malnutrition risk (40–45), or severe malnutrition risk (PNI < 40).

Both indices were calculated using laboratory values obtained within the first 24 h of admission, prior to reperfusion therapy or any nutritional intervention. The association between these nutritional indices and in-hospital mortality was subsequently analyzed.

### 2.2. Data Collection

Clinical, demographic, and laboratory characteristics were obtained from patient files and computerized hospital records. Baseline parameters, including urea, creatinine, total cholesterol, glucose, and C-reactive protein (CRP) levels, as well as troponin-I, were measured before P-PCI. Total protein and albumin concentrations were determined photometrically (DC 800; Beckman Coulter, Dublin, Ireland) within the first 24 h after admission. Troponin-I levels were measured every 6 h until the peak value and daily thereafter. B-type natriuretic peptide (BNP) was determined by immunoassay (ADVIA Centaur; Bayer, Tarrytown, NY, USA).

Standard 12-lead ECGs were obtained on admission, immediately after the procedure, at 60 min, and periodically thereafter. Post-procedural transthoracic echocardiography (Vivid 5; GE Vingmed Ultrasound AS, Horten, Norway) was performed during hospitalization, and left ventricular ejection fraction (LVEF) was calculated using the biplane Simpson’s method.

All patients received standard dual antiplatelet therapy with 300 mg aspirin and 600 mg clopidogrel loading doses, as well as 70 U/kg intravenous heparin prior to the procedure.

P-PCI was performed by experienced interventional cardiologists via the femoral approach. Culprit lesions were crossed with 0.014-inch guidewires, and direct or conventional stenting, or balloon angioplasty, was performed according to lesion morphology. Final Thrombolysis in Myocardial Infarction (TIMI) flow grade and myocardial blush grade (MBG) were determined by established criteria.

### 2.3. Statistics

Baseline clinical, demographic, and laboratory characteristics were compared between patients with and without in-hospital mortality. Continuous variables were first assessed for normality using the Shapiro–Wilk test. Normally distributed continuous variables are presented as mean ± standard deviation and were compared using the independent samples *t*-test. Non-normally distributed continuous variables are expressed as median with interquartile range and were compared using the Mann–Whitney U test. Categorical variables are presented as frequencies and percentages and were compared using the chi-square test or Fisher’s exact test, as appropriate. A two-sided *p*-value < 0.05 was considered statistically significant.

The primary outcome was in-hospital mortality. Candidate predictors were selected based on biological plausibility and prior evidence of association with all-cause mortality, including age, sex, diabetes mellitus (DM), smoking, previous myocardial infarction (MI), Killip class, BMI, total ischemic time, hemoglobin, systolic blood pressure, heart rate, platelet count, creatinine, previous statin use, and no-reflow phenomenon.

To reduce the risk of overfitting, the number of covariates included in the multivariable models was guided by clinical relevance and prior literature. Multicollinearity was assessed using variance inflation factors (VIF), and no significant collinearity was detected.

Three multivariable logistic regression models were constructed:

Model 1 (baseline model): included established clinical and laboratory predictors of in-hospital mortality defined as above.

Model 2: Model 1 plus the Prognostic Nutritional Index (PNI).

Model 3: Model 1 plus the Controlling Nutritional Status (CONUT) score.

Statistical modeling: Binary logistic regression was used to explore the association between nutritional indices and in-hospital mortality. Non-linear relationships were modeled using cubic splines. For continuous predictors, odds ratios (ORs) represented the change from the 25th to the 75th percentile. Variables with >20% missing data were excluded, and multiple imputation (five datasets) was applied under the missing-at-random assumption; Rubin’s rule was used to pool estimates.

Model performance: Model goodness of fit was assessed using the likelihood ratio chi-square (LR χ^2^), Akaike information criterion (AIC), and Bayesian information criterion (BIC). Discrimination was evaluated with the receiver operating characteristic area under the curve (ROC-AUC), and model improvement was tested with the continuous net reclassification index (NRI) and integrated discrimination improvement (IDI). Statistical analyses were conducted using R software version 4.0.1 (Vienna, Austria).

## 3. Results

The median age of the study population (*n* = 4599) was 58 years, and 41.1% of patients were female. Among the included patients, 53.3% had anterior myocardial infarction, 25% had DM, and the no-reflow phenomenon was observed in 16.2%. Other baseline demographic and clinical characteristics are summarized in Table 1.

The overall in-hospital mortality rate was 5.7% (*n* = 261). Patients who died during the index hospitalization were significantly older and had higher rates of DM, previous MI, and prior revascularization. They also exhibited longer total ischemic times, more frequent no-reflow, and lower LVEF compared with patients without in-hospital mortality (Table 1).

**Figure 2:**
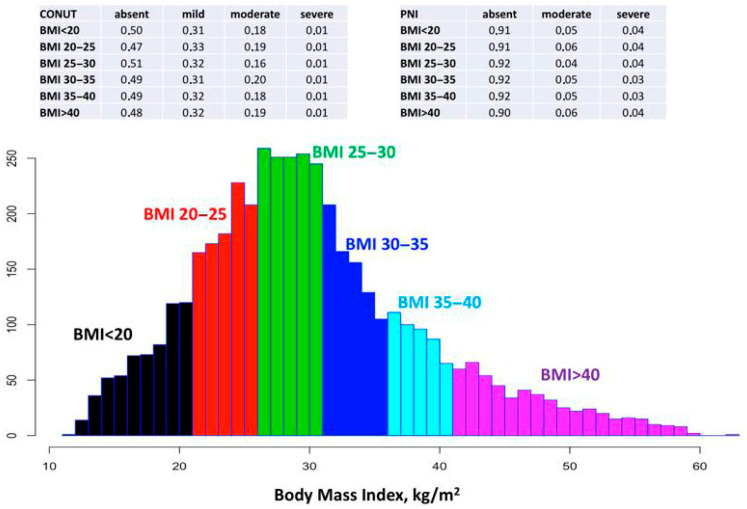
Distribution of body mass index (BMI) and corresponding malnutrition frequency. BMI, body mass index; CONUT, Controlling Nutritional Status Monitor; PNI, The Prognostic Nutritional Index.

The median PNI was 46.5 (42.3–51.0) and CONUT was 2.0 (0.0–3.0). When malnutrition was assessed using the CONUT score, 49.4% of patients were classified as having normal nutritional status, while 31.9%, 18.0%, and 0.7% had mild, moderate, and severe malnutrition, respectively. In contrast, when the PNI was used to evaluate nutritional status, 91.1% of patients were classified as normal, and 5.1% and 3.8% had moderate and severe malnutrition, respectively. Across BMI categories, the prevalence of moderate-to-severe malnutrition ranged between 17–21% and 8–10% when assessed by CONUT and PNI, respectively. Further details are illustrated in Figure 2.

**Figure 3:**
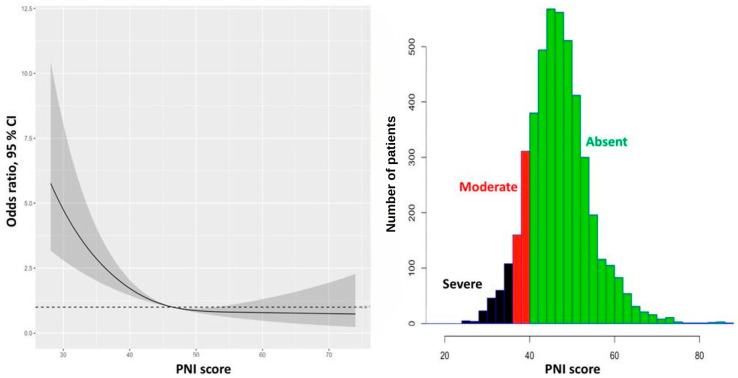
Distribution of the Prognostic Nutritional Index (PNI) in the study population (histogram, left axis) and adjusted odds ratios for in-hospital mortality across PNI values derived from multivariable logistic regression with restricted cubic splines (solid line). Shaded areas represent 95% confidence intervals. Odds ratios are adjusted for age, sex, diabetes mellitus, previous myocardial infarction, Killip class, total ischemic time, hemoglobin, creatinine, systolic blood pressure, heart rate, platelet count, previous statin use, and no-reflow phenomenon. CI, confidence interval; PNI, the Prognostic Nutritional Index. The dashed horizontal line represents an odds ratio of 1.

**Figure 4:**
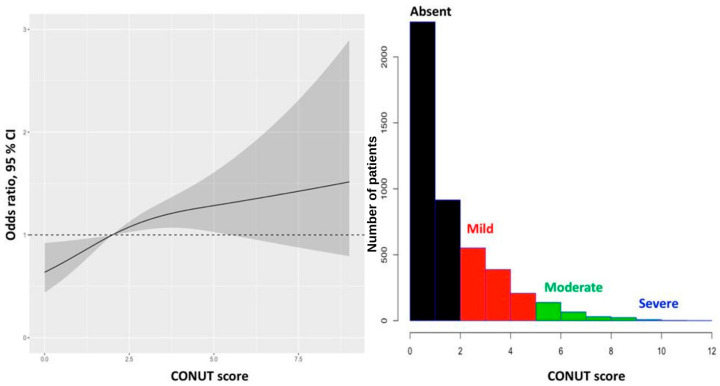
Distribution of the Controlling Nutritional Status Monitor (CONUT) score in the study population (histogram, left axis) and adjusted odds ratios for in-hospital mortality across CONUT values derived from multivariable logistic regression with restricted cubic splines (solid line). Shaded areas represent 95% confidence intervals. Odds ratios are adjusted for age, sex, diabetes mellitus, previous myocardial infarction, Killip class, total ischemic time, hemoglobin, creatinine, systolic blood pressure, heart rate, platelet count, previous statin use, and no-reflow phenomenon. CI, confidence interval; CONUT, Controlling Nutritional Status Monitor. The dashed horizontal line represents an odds ratio of 1.

We first constructed a baseline multivariable logistic regression model without nutritional indices (Model 1). Subsequently, two additional models were developed: one incorporating the Prognostic Nutritional Index (Model 2) and the other incorporating the Controlling Nutritional Status score (Model 3). Both indices showed a statistically significant and independent association with in-hospital mortality in their respective models (Figure 3 and Figure 4, and Table 2).

The model that included CONUT (Model 3) demonstrated superior predictive performance compared with both the baseline model (Model 1) and the model incorporating the PNI score (Model 2). Model 3 exhibited the lowest AIC and BIC values, the highest LR χ^2^, and the greatest discriminative ability among all models (Table 3).

Furthermore, both models incorporating the CONUT and PNI indices demonstrated a significantly greater incremental prognostic value for predicting in-hospital mortality compared with the baseline model (Table 4).

## 4. Discussion

Our findings indicate that malnutrition, as assessed by either the PNI or the CONUT score, serves as a significant prognostic marker in patients with STEMI. Moreover, the prognostic performance of the CONUT was superior to that of the PNI score.

Several biological mechanisms may explain the association between malnutrition and adverse outcomes in STEMI. Malnutrition is closely linked to systemic inflammation, impaired immune response, reduced metabolic reserve, and sarcopenia, all of which may contribute to delayed tissue repair, impaired myocardial healing, and increased vulnerability to complications during the acute phase of myocardial infarction [20,21].

Both the PNI and CONUT reflect not only nutritional status but also systemic inflammation. Cheng et al. demonstrated that PNI was independently associated with long-term survival among hospitalized patients with worsening heart failure, regardless of LVEF status [21]. As the PNI is derived from serum albumin levels and lymphocyte counts, it reflects both nutritional reserve and immune competence. Anker et al. reported that nearly 50% of patients with advanced heart failure were malnourished, and approximately 15% fulfilled the criteria for cachexia [22]. Cardiovascular cachexia, characterized by increased resting metabolic rate and gastrointestinal malabsorption, represents a severe catabolic state associated with poor outcomes [23,24].

Malnutrition is closely linked to chronic inflammation, which contributes to increased atherosclerotic burden. The interplay of these factors has recently been termed the malnutrition–inflammation–atherosclerosis (MIA) syndrome [25,26]. Prior studies in patients with heart failure showed that indices of malnutrition also mirrored inflammatory activity and disease severity, both of which significantly impact prognosis [27].

Acute coronary syndromes (ACS) remain a major cause of mortality and morbidity. Therefore, identifying early predictors of in-hospital and long-term mortality is of great clinical importance. Several risk scores, such as TIMI, Age, Creatinine, and Ejection Fraction Score (ACEF) and Global Registry of Acute Coronary Events Score (GRACE), are routinely used for risk stratification in this context [28,29,30,31]. Additionally, lower serum albumin concentrations and indicators of malnutrition have been shown to predict poor prognosis [32]. Roubin et al. reported that malnutrition was present in up to 50–60% of patients presenting with ACS, independent of BMI [4]. Notably, approximately 40% of overweight or obese individuals by BMI criteria were malnourished, highlighting that excess body weight does not preclude poor nutritional status. Chronic inflammatory states may increase catabolic cytokines, suppress appetite, and promote muscle catabolism, resulting in reduced albumin levels. Similarly, in our study, malnutrition was observed across all BMI categories and was associated with an increase in in-hospital mortality. The present analysis, which included the largest cohort of STEMI patients to date, demonstrated that both PNI and CONUT were significant predictors of short-term mortality. In a study involving 908 patients aged over 70 years, Tonet et al. reported that 44% of elderly ACS patients were malnourished or at risk of malnutrition using the Mini Nutritional Assessment–Short Form [33].

Previous research often examined PNI and CONUT individually as indicators of nutritional and inflammatory status, but few studies compared their prognostic performance in STEMI. In our cohort, CONUT demonstrated greater predictive accuracy than PNI. This difference may be explained by the components of each index. The PNI incorporates only serum albumin and lymphocyte count, while the CONUT additionally includes total cholesterol. Although the CONUT score theoretically captures hypocholesterolemia as a marker of malnutrition, widespread statin therapy in modern cardiac populations may attenuate this relationship. Oduncu et al. showed that lowering LDL-cholesterol with statins improved outcomes in STEMI patients [34]. Approximately 20% of our cohort were on statin therapy, which may partly explain the observed differences between CONUT and PNI in predictive performance.

Unlike prior studies that evaluated CONUT or PNI separately, the present study directly compares both indices within the same large STEMI cohort using comprehensive model performance metrics, including discrimination, calibration, and reclassification analyses. This approach allows a more nuanced assessment of their relative prognostic utility in contemporary STEMI patients undergoing P-PCI.

Based on these findings, the CONUT may be a simple, objective, and cost-effective marker that can be integrated into early risk assessment in STEMI. Beyond prognostication, improving nutritional status may itself have therapeutic benefits. A recent randomized clinical trial demonstrated that individualized nutritional support in hospitalized patients at nutritional risk improved survival and key clinical outcomes [35]. Dietary counseling, educational programs, oral nutritional supplements, and food/fluid fortification or enrichment have all been established as interdisciplinary techniques for preventing and treating malnutrition [36]. Moreover, when combined with additional extensive lifestyle adjustments, dietary therapies have been associated with regression of coronary atherosclerosis [37]. These data collectively emphasize the importance of nutritional optimization in cardiovascular care.

In addition to biochemical and nutritional indices, contemporary risk stratification in STEMI increasingly incorporates advanced imaging modalities such as cardiac magnetic resonance. Tissue characterization with late gadolinium enhancement and assessment of the remote myocardium have been shown to provide incremental prognostic information beyond traditional clinical parameters. Integration of nutritional status with advanced imaging markers may represent a promising future direction for comprehensive risk stratification in STEMI [38].

Several limitations should be acknowledged. First, this was a retrospective, single-center study, which limits causal inference and may introduce selection bias. Second, the analysis was restricted to in-hospital mortality, and causes of death (cardiac vs. non-cardiac) could not be distinguished. Third, follow-up data after hospital discharge were not available, precluding assessment of long-term prognostic implications. Fourth, although baseline variables were selected based on clinical relevance and prior literature, residual confounding cannot be excluded. Finally, given the number of predictors included relative to the number of outcome events, the possibility of model overfitting should be considered, despite the use of model performance and validation metrics.

Despite these limitations, this study has several notable strengths, including a large and homogeneous STEMI population, the simultaneous evaluation of two widely used nutritional indices, and the use of easily accessible routine laboratory parameters. Together, these features enhance the clinical applicability and generalizability of our findings. However, future multicenter, prospective studies with serial nutritional assessments and long-term follow-up are warranted to validate these results and to clarify whether nutritional optimization can translate into improved clinical outcomes in STEMI.

## 5. Conclusions

Among patients with STEMI, moderate-to-severe malnutrition was present in approximately 10% and 20% of patients when evaluated using the PNI and CONUT scores, respectively. In terms of in-hospital mortality, CONUT may provide incremental prognostic information compared with PNI; however, given the retrospective design and lack of long-term follow-up, these findings warrant confirmation in prospective studies.

## Figures and Tables

**Table 1:** Baseline characteristics of the study population.

	Overall (*n* = 4599)	Mortality (−) (*n* = 4338)	Mortality (+) (*n* = 261)	*p* Value
**Gender, female, n (%)**	1888 (41.1)	1775 (40.9)	113 (43.3)	0.492
**Age, year**	58 (50–67)	58 (50–67)	67 (56–78)	<0.001
**DM, n (%)**	1146 (24.9)	1050 (24.2)	94 (36.0)	<0.001
**HT, n (%)**	2062 (44.8)	1924 (44.4)	136 (52.1)	0.017
**Current smoker, n (%)**	2466 (53.6)	2361 (54.5)	103 (39.5)	<0.001
**Hyperlipidemia, n (%)**	1316 (28.6)	1264 (29.2)	52 (19.9)	0.002
**Family history of CAD, n (%)**	1056 (23.1)	1024 (23.7)	32 (12.6)	<0.001
**Previous MI, n (%)**	685 (14.9)	619 (14.3)	64 (24.5)	<0.001
**Previous revascularization, n (%)**	679 (14.8)	618 (14.3)	59 (22.6)	<0.001
**Height, cm**	172 (162–183)	172 (163–183)	169 (160–178)	<0.001
**Weight, kg**	86 (70–106)	86 (71–107)	82 (68–99)	0.016
**BMI, kg/m^2^**	28.8 (24.0–34.6)	28.8 (24.0–34.6)	28.8 (24.0–34.6)	0.834
**Anterior MI, n (%)**	2450 (53.3)	2331 (53.8)	118 (45.2)	0.009
**Pain to door time, min**	142.5 (75–240)	140 (75–240)	150 (80–290)	0.022
**Door to balloon time, min**	30 (26–35)	30 (25–35)	32 (27–90)	<0.001
**Total ischemic time, min**	183 (120–297)	182 (120–291)	211 (135–360)	<0.001
**Killip II-III-IV, n (%)**	675 (14.7)	576 (13.3)	98 (37.5)	<0.001
**Systolic blood pressure, mmHg**	132 (117–145)	132 (118–146)	120 (90–140)	<0.001
**Diastolic blood pressure, mmHg**	78 (68–87)	78.0 (68.0–87.8)	70 (52–80)	<0.001
**Heart rate, beats/min**	79 (69–89)	79 (69–88)	87 (77–103)	<0.001
**White blood cell (cells/µL)**	11.8 (9.7–14.2)	11.7 (9.70–14.1)	13.2 (11.1–16.8)	<0.001
**Neutrophil count (cells/µL)**	9.0 (7.0–11.4)	8.95 (6.99–11.2)	10.7 (8.6–14.3)	<0.001
**Lymphocyte count (cells/µL)**	1.75 (1.25–2.40)	1.79 (1.28–2.40)	1.41 (1.05–2.01)	<0.001
**Hemoglobin (g/dL)**	13.8 (12.5–14.9)	13.9 (12.5–14.9)	12.5 (10.9–14.1)	<0.001
**Platelet count (/mm^3^)**	251 (211–298)	251 (211–297)	258 (215–307)	0.214
**Creatinine (mg/dL)**	0.87 (0.77–1.02)	0.87 (0.77–1.02)	0.97 (0.80–1.40)	<0.001
**Blood glucose (mg/dL)**	130 (107–175)	129 (107–174)	148 (119–225)	<0.001
**Albumin (g/dL)**	3.70 (3.50–4.00)	3.80 (3.50–4.00)	3.40 (3.20–3.70)	<0.001
**C-reactive protein (mg/L)**	7.71 (2.52–14.1)	7.70 (2.50–14.1)	8.6 (3.06–13.6)	0.184
**Total-cholesterol (mg/dL)**	177 (151–202)	177 (151–202)	170 (148–205)	0.259
**LDL-cholesterol (mg/dL)**	112 (88–136)	112 (88–136)	112 (86–141)	0.743
**HDL-cholesterol (mg/dL)**	36 (30–43)	36.1 (30.7–43)	36 (28–43)	0.100
**Triglyceride (mg/dL)**	125 (91–170)	125 (91–170)	115 (88–162)	0.025
**Left ventricular EF (%)**	47 (40–53)	48 (40–54)	38 (30–44)	<0.001
**Stent length, mm**	20 (18–28)	20 (18–28)	22 (18–31)	0.010
**Stent diameter, mm**	3.00 (2.75–3.25)	3.00 (2.75–3.25)	3.00 (2.75–3.00)	0.078
**Baseline TIMI flow, n (%)**				
** 0**	3120 (69.3)	2935 (68.4)	185 (87.7)	
** 1**	551 (12.2)	540 (12.6)	11 (5.2)	<0.001
** 2**	504 (11.2)	493 (11.5)	11 (5.2)	
** 3**	326 (7.2)	321 (7.5)	4 (1.9)	
**Final TIMI flow, n (%)**				
** 0**	131 (2.9)	100 (2.3)	31 (14.7)	
** 1**	173 (3.8)	133 (3.1)	40 (19.0)	<0.001
** 2**	425 (9.4)	380 (8.9)	45 (21.3)	
** 3**	3772 (83.8)	3676 (85.7)	95 (45.0)	
**Previous medications, n (%)**				
** Beta-blocker**	547 (12.1)	507 (11.8)	40 (16.5)	0.040
** Statin**	760 (16.5)	707 (16.3)	51 (19.5)	0.200
** ACE-I/ARB**	945 (20.5)	884 (20.4)	59 (22.6)	0.434
** Antiplatelet agents**	659 (14.6)	617 (14.4)	42 (17.3)	0.251

ACE-I/ARB: Angiotensin converting enzyme inhibitors/angiotensin receptor blockers; BMI: body mass index; CAD, coronary artery disease; DM, diabetes mellitus; EF, ejection fraction; HDL, high-density lipoprotein; HT, hypertension; LDL, low-density lipoprotein; MI, myocardial infarction; TIMI, the thrombolysis in myocardial infarction score.

**Table 2:** Multivariable logistic regression analyses of Controlling Nutritional Status (CONUT), The Prognostic Nutritional Index (PNI) scores and in-hospital mortality.

Models	Odds Ratio (95% CI)	*p* Value
**Model with CONUT, risk of malnutrition** CONUT score 1 (absent) CONUT score 5 (mild) CONUT score 7 (moderate) CONUT score 10 (severe)	Ref. 1.57 (1.19–2.10) 1.71 (1.11–2.64) 1.93 (0.93–4.01)	0.005
**Model with PNI, risk of malnutrition** PNI score 45 (absent) PNI score 37 (moderate) PNI score 30 (severe)	Ref. 2.11 (1.64–2.72) 4.29 (2.59–7.11)	<0.001

**Table 3:** Comparative analyses of goodness of fit and model performances.

	LR χ^2^	AIC	BIC	R-Square	AUC
**Model 1**	453	1601	1762	0.266	0.831
**Model 2**	464	1598	1784	0.272	0.841
**Model 3**	492	1570	1757	0.287	0.855

AIC, Akaike Information Criterion; AUC, Area Under the Curve; BIC, Bayesian Information Criterion; LR χ^2^, Likelihood Ratio Chi-Square.

**Table 4:** Comparison of models by continuous net reclassification improvement (NRI) and integrated discrimination improvement (IDI).

	Continuous NRI	*p* Value	IDI	*p* Value
**Model 1 vs. 2**	0.239	0.001	0.001	0.729
**Model 1 vs. 3**	0.329	<0.001	0.012	0.024
**Model 2 vs. 3**	0.124	0.050	0.011	0.009

IDI, integrated discrimination improvement; NRI, net reclassification improvement.

## Data Availability

The data that support the findings of this study are available from the corresponding author upon reasonable request. Due to patient privacy and institutional regulations, the data are not publicly available. Artificial intelligence-assisted technologies, chatbots, or image creators during the production of the submitted work were not used in this study.
